# Social Media Marketing Strategies for Electronic Cigarettes: Content Analysis of Chinese Weibo Accounts

**DOI:** 10.2196/51594

**Published:** 2024-11-07

**Authors:** Xinyi Zhou, Xinyu Hao, Yuhang Chen, Hui Deng, Ling Fang, Lingyun Zhang, Xiaotao Yan, Pinpin Zheng, Fan Wang

**Affiliations:** 1 Fudan Development Institute Fudan University Shanghai China; 2 Asia Research Center Fudan University Shanghai China; 3 School of Politics and International Relations East China Normal University Shanghai China; 4 School of Journalism Fudan University Shanghai China; 5 Department of Preventive Medicine and Health Education School of Public Health Fudan University Shanghai China; 6 Key Laboratory of Public Health Safety Fudan University Shanghai China

**Keywords:** e-cigarette, marketing strategy, social media, teenagers, content analysis

## Abstract

**Background:**

E-cigarettes have gained popularity among teenagers due to extensive marketing strategies on social media platforms. This widespread promotion is a risk factor, as it fosters more positive attitudes toward e-cigarette use among teenagers and increases the perception that using e-cigarettes is normal. Therefore, the marketing of e-cigarettes on social media is a serious global health concern, and its strategies and impact should be clearly identified.

**Objective:**

This study examined how e-cigarette companies popularize their products via Weibo and identified the specific strategies influencing the effectiveness of their marketing.

**Methods:**

In phase 1, we conducted a search on Qcc.com and identified 32 e-cigarette brands with active Weibo accounts between October 1 and December 31, 2020, along with 863 Weibo posts. The data were investigated through content analysis. The codebook was developed into four categories: (1) product and features, (2) sales and promotions, (3) social contact and interaction, and (4) restrictions and warnings. To further understand the factors influencing e-cigarette brand marketing, we conducted a multiple linear regression analysis.

**Results:**

Marketing tactics by e-cigarette companies on Chinese social media were documented, including emphasizing attractive product features, using trendy characters, implicit promotions, downplaying health concerns, and engaging with Weibo users in various ways. Out of 863 posts, 449 (52%) mentioned product characteristics. In 313 (36.3%) posts, visible figures were used to attract attention. Product promotion was absent in 762 (88.3%) posts, and purchase channels were not mentioned in 790 (98.3%) posts. Social interaction–related posts received attention (n=548, 63.5%), particularly those featuring hashtag content (n=538, 62.3%). Most posts did not include claims for restrictions on teenagers' purchases or use (n=687, 79.6%) or information on health warnings (n=839, 97.2%). Multiple linear regression analysis identified marketing strategies that effectively increase the exposure of e-cigarette posts on Weibo. Posts including engagement via posts encouraging reposts, comments, and likes (*P<.*001) and engagement topics related to e-cigarette brands were positively correlated with the number of reposts (*P=.*009). Posts highlighting nonmonetary incentives (*P=.*004), posts with age restriction statements (*P<.*001), engaging via stories and idea collection (*P<.*001), and engagement topics related to products (*P<.*001) and current affairs (*P=*.002) had a positive effect on the number of comments. Engagement topics related to brands (*P*<.001) or interactive sweepstakes (*P*<.001) had a positive effect on the number of likes.

**Conclusions:**

E-cigarette posts on Weibo that focus on product features and social interaction attract public attention, especially from teenagers. Stricter regulations and monitoring should be adopted to restrict the social media marketing of e-cigarettes.

## Introduction

The prevalence of emerging nicotine and tobacco products, including electronic nicotine delivery systems (ENDS), has significantly increased worldwide [1], posing serious health concerns. E-liquids created by e-cigarettes contain toxic and potentially carcinogenic metals, increasing the likelihood of developing chronic health issues [2,3]. However, e-cigarettes have emerged as the most prevalent tobacco product among teenagers worldwide [4,5]. According to the US Centers for Disease Control, in 2022, over 2.55 million middle and high school students reported e-cigarette use within the past 30 days in the United States [6]. In China, the 2021 National Tobacco Prevalence Survey of High School and College Students reported that 86.6% of middle school students were aware of e-cigarettes, while 3.6% had used them. These figures represent a 9.2% and 0.8% increase, respectively, compared with 2019 [7]. In many countries, e-cigarette use rates among young people are higher than those among adults [8]. The e-cigarette trend prompted the World Health Organization (WHO) to strictly regulate ENDS to ensure the maximum protection of public health [1].

An increasing number of published studies have demonstrated that the popularity of e-cigarettes is largely attributed to their extensive propagation on social media platforms [9-11]. Notwithstanding this, e-cigarette marketing on social media is not closely monitored [12,13], likely contributing to a surge in the marketing and consumption of these products. For example, currently, in the United States, e-cigarette companies not bound by the Tobacco Master Settlement Agreement, an agreement that has committed to reducing smoking rates among Americans, can promote their products on social media platforms such as Twitter, Facebook, Instagram, and YouTube [14-16]. In the early years, a similar regulatory gap existed in China [17]. Since 2020, the Chinese government has recognized the potential health hazards of e-cigarettes, particularly for teenagers [18]. The Special Inspection Action Plan for the E-cigarette Market was implemented, which conducted a comprehensive clean-up of e-cigarette–related information on the internet [19]. Since 2021, the State Tobacco Monopoly Administration in China has the authority to supervise new tobacco products, including e-cigarettes [20], with stricter regulatory oversight.

Nevertheless, the implicit promotion of e-cigarettes on specific social media platforms has persisted in China for a considerable period. This promotion is driven by both e-cigarette companies and users, aiming to make the product more appealing through attractive flavors [12], eye-catching packaging design [13,21], and positioning it as a cessation tool [22]. Similar challenges are faced in Australia, the United States, the United Kingdom, and Indonesia [23-25]. Exposure to promotional content related to e-cigarettes on social media is identified as a risk factor contributing to heightened intentions to use e-cigarettes, more favorable attitudes toward e-cigarettes, and an increased perception of e-cigarette use as a normative behavior, particularly among teenagers [26,27].

Weibo is China’s most popular social media platform, integrating the features of Twitter, YouTube, and Instagram. In 2020, 30% of Weibo active users were born after 2000, indicating that teenagers are an important user group [28]. Teenagers use Weibo for social interaction, self-presentation, and access to various types of information, including entertainment gossip, social news, brand marketing, and other types of information [29]. Given that most teenagers engage with numerous social media platforms several times per day [30], they are exposed to a considerable quantity of e-cigarette information on social media, are particularly vulnerable to such messaging, and have demonstrated considerable interest in vaping [14,31,32]. This expands the accessibility of e-cigarette information, influencing the likelihood of e-cigarette use and shaping social norms around e-cigarette use among young people [1].

Given the growing apprehension regarding the widespread appeal of e-cigarette–related content and the potential to cultivate a new generation of nicotine-dependent users [33], particularly among teenagers, examining Weibo posts on how e-cigarette companies have popularized their products is meaningful. The results can inform policy makers on how e-cigarettes are being promoted and marketed on social media, which is critical for intensifying regulations around e-cigarettes. To address these concerns, we aimed to (1) investigate the characteristics of marketing strategies of e-cigarette advertisements on Weibo and (2) examine the factors influencing the perceived attractiveness of e-cigarettes on Weibo.

## Methods

### Aim 1: Marketing Strategies of E-Cigarette Advertisements on Weibo

#### Data Collection

First, we conducted a search on the Chinese enterprise search website Qcc.com [34] using the keyword “e-cigarettes” with specific constraints such as “industry category=manufacturing,” “search scope=company name/brand/product,” and “registration status=current/existing.” This search yielded a total of 3746 electronic cigarette companies. Second, we performed a data cleansing process in which we excluded 2403 non–e-cigarette companies that were unrelated to e-cigarettes or had names such as Electronic Commerce Centre, Experience Store, Authorized Store, Studio, and similar contents. Next, we examined the remaining dataset and excluded 1239 companies that lacked a company website or had an inaccessible website. As a result, we obtained a final list of 104 e-cigarette companies. Third, using the names and relevant information of these 104 e-cigarette companies, we searched on Weibo and found 181 related Weibo accounts. Of these 181 accounts, 66 (36.5%) had updated Weibo posts in 2020 and 32 (17.7%) posted new content during our research period (October 1 to December 31, 2020). Finally, we selected 32 e-cigarette brands and their official Weibo accounts to include in our research.

Our analysis included all 863 posts published by the 32 accounts from October 1, 2020, to December 31, 2020. All the posts were scraped by researchers manually. The sample collection was completed in early January 2021.

#### Developing the Coding Framework

We reviewed the existing studies on e-cigarette social media marketing and browsed through basic account information and all 863 posts from 32 e-cigarette Weibo accounts to develop the coding framework. First, we reviewed previous relevant content analysis and summarized important strategies to test in our study [1] [35-46]. Second, we reviewed the 863 posts, combined the characteristics of Weibo, and created an initial codebook comprising four analysis categories: (1) product and features [22,36], (2) sales and promotion [23,37], (3) social contact and interaction, and (4) restrictions and warnings [47,48]. The coding framework was established through discussions and revisions among the researchers involved in this study. We entered the data into EpiData (EpiData Association) via the double-entered method and imported it into SPSS (version 29.0; IBM Corp) for data analysis. To examine the characteristics of the Weibo posts, we conducted a descriptive analysis by calculating the frequency and percentage distribution of each item in the codebase.

### Aim 2: Examining Factors Influencing the Attractiveness of E-Cigarettes on Weibo

The codebook includes all independent variables for the analysis of influencing factors. To evaluate whether the items of the Weibo post characteristics in our codebook could influence the media effect of e-cigarette–related posts collected in our study, we used the Spearman bivariate correlation test to screen independent variables that were correlated with the numbers of reposts, comments, and likes. Then, we conducted 3 multiple linear regression analyses based on the number of reposts, comments, and likes. These analyses helped us identify the independent variables to be included in the final model. We used coefficient B (B) to indicate the amount of change in the dependent variables for one unit change in the number of reposts, comments, and likes. The SE measured the uncertainty of the regression coefficient estimates across the 3 models. We used standardized coefficient beta (β) to allow for a direct comparison of the relative influence of different variables in the regression analysis. T-value (T) tested the significance of the influence of independent variables on dependent variables. Variance inflation factor (VIF) detected multicollinearity between independent variables. A VIF of less than 10 is considered not to affect the model’s stability. We conducted tests of statistical significance using *P*<.05 as a threshold to report statistical significance.

### Ethical Considerations

This study observed the marketing strategies of e-cigarette companies and public behaviors on social media. This study did not involve human participants, as all data were acquired from public social media platforms. All the names of e-cigarette brands and users participating in the Weibo interaction are anonymous. Therefore, no additional ethical review was necessary.

## Results

### Sample of Posts

**Figure 1:**
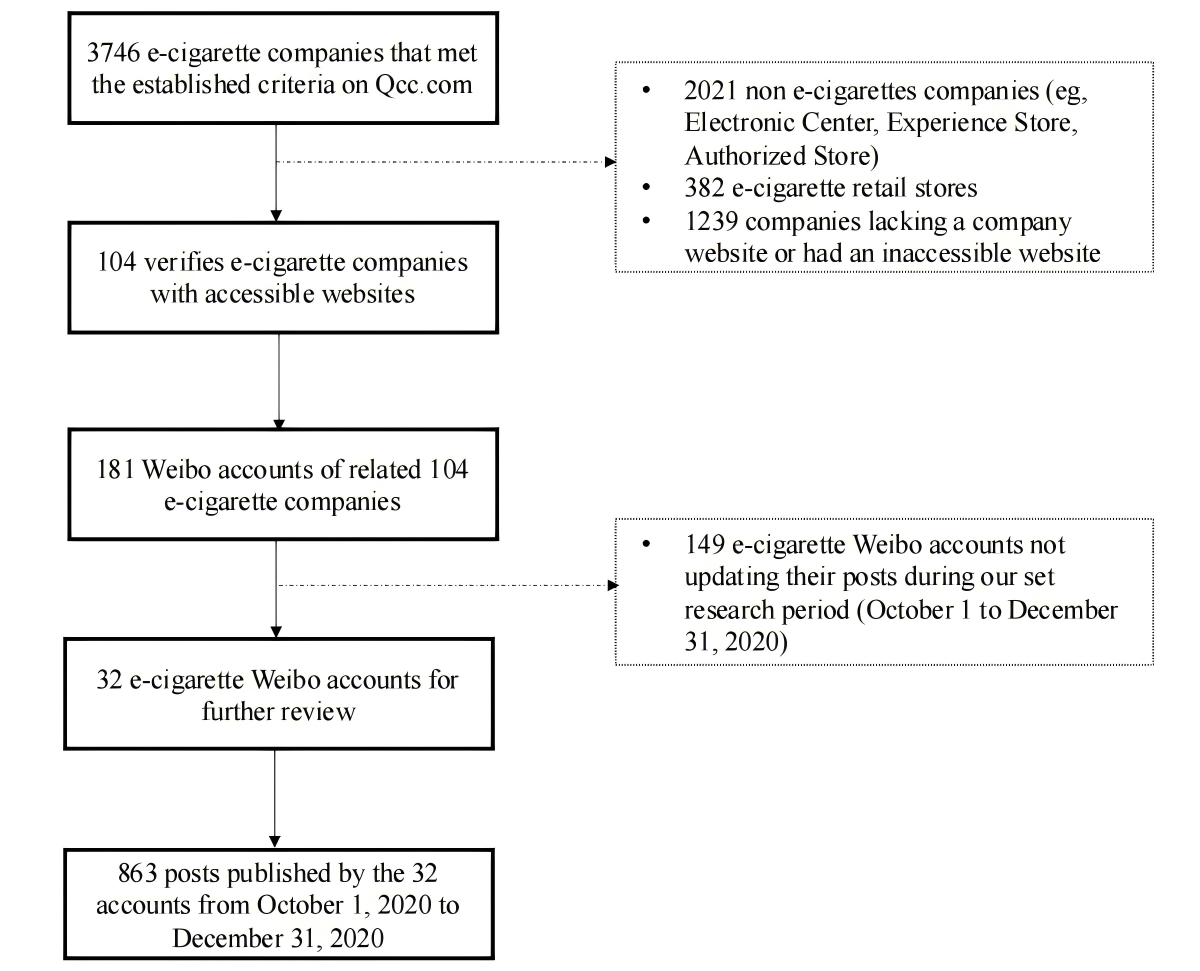
PRISMA (Preferred Reporting Items for Systematic Reviews and Meta-Analyses) checklist that this study followed.

Figure 1 shows this study’s screening process, which followed the PRISMA (Preferred Reporting Items for Systematic Reviews and Meta-Analyses) checklist. Multimedia Appendix 1 provides descriptive statistics for the characteristics of 863 posts from 32 selected Chinese e-cigarette Weibo accounts. Unlike traditional media, social media platforms facilitate individual interactions through comments and sharing. The number of reposts, comments, and likes reflected how e-cigarettes were perceived. Of the 863 Weibo posts, there were a total of 24,571 reposts, 8516 comments, and 22,272 likes. The average numbers of reposts, comments, and likes were 28.47 (SD 286.13), 9.87 (SD 41.27), and 25.81 (SD 237.63), respectively. Among these posts, the highest number of reposts, comments, and likes were 4903 (20%), 677 (7.9%), and 5029 (22.56%), respectively. The top 3 posts with the most reposts, comments, and likes all included interactive sweepstakes using e-cigarette and comparable products.

### Product and Features

As Multimedia Appendix 1 shows, of the 863 Weibo posts, 449 (52%) posts described details of product features to attract others, including 351 (40.7%) posts relating to the shape of e-cigarettes and packaging attractiveness, 111 (12.9%) posts about flavors, and 48 (5.6%) posts highlighting the high quality and technique used in e-cigarettes. Out of 863 posts, 313 (36.3%) contained visible figures and 81 (9.4%) depicted fashion icons. Other visible characters with the highest percentage were everyday people (n=78, 9%), and the remaining were business people (n=26, 3%), sports people (n=16, 1.9%), celebrities (n=13, 1.5%), sales agents (n=12, 1.4%), and health professionals (n=2, 0.2%).

### Sales and Promotions

A total of 101 (11.7%) posts had information on product promotions. Among them, 10 (1.2%) posts involved monetary promotional offers, including coupons, two-for-one offers, and discount sales. Moreover, 83 (9.6%) posts were related to nonmonetary promotions including giveaways and free samples, while 8 (0.9%) posts contained both incentives.

A total of 790 (91.5%) Weibo posts did not mention any information on purchase channels. The rest (n=73, 8.5%) still contained information on purchase links, phone numbers of offline purchases, and other purchase channels.

### Social Contact and Interaction

Of the 863 posts, 548 (63.5%) interacted with fans, indicating that most posts tried to entice Weibo users to engage through various methods, including reposting, commenting, and clicking likes. A total of 538 (62.3%) posts featured hashtags; 41 (4.8%) encouraged fans to repost, comment, and like; and 29 (3.4%) interacted with fans by collecting stories and ideas.

E-cigarette–related posts generated interactions with Weibo users through a variety of topics. In 45.4% (n=392) of the posts, interaction topics were related to products. Current event topics accounted for 21.1% (n=182) of the posts. Topics related to interactive sweepstakes accounted for 8.7% (n=75). The post number of brand-related topics, “soul-soother” or emotional issues, and Weibo account promotion were 73 (8.5%), 15 (1.7%), and 1 (0.1%), respectively. Furthermore, joint promotion with other brands and promoting the Weibo accounts of brands by sending gifts were also the main themes of hashtag topics.

### Restrictions and Warnings

Of the posts, 687 (79.6%) posts had no age restriction claims for minors to purchase or use e-cigarettes, 130 (15.1%) restricted minors’ use but did not limit their purchase, and 6 (0.7%) restricted minors’ purchases but did not limit their use. Only 40 (4.6%) posts restricted both purchases and use by minors. Meanwhile, only 24 (2.8%) posts provided health warnings.

We put all the variables in the codebook into the Spearman bivariate correlation test to screen out the independent variables that had a significant correlation with the dependent variable. These variables were included as independent variables in multiple linear regression. A multiple linear regression analysis was conducted to examine the effect of e-cigarette postmarketing strategy features on communication outcomes. The overall statistics of the 3 regression models are presented in Table 1. The results are presented in Table 2.

**Table 1:** Summary statistics of multiple linear regression models.

Models	Dependent variables	*R* ^2^	*F* test (*df*)	*P* value
Model 1	Number of reposts	0.183	9.224 (13)	<.001
Model 2	Number of comments	0.211	11.935 (12)	<.001
Model 3	Number of likes	0.204	10.512 (12)	<.001

**Table 2:** Results of multiple linear regression analysis for the influence of Weibo marketing strategies on the number of reposts, comments, and likes in China (N=863).

Model			Nonstandardized coeffificients				Standard coeffificients		T^a^		*P* value		VIF^b^
			B^c^		SE		Β^d^						
**Model 1**													
	Constant	–23.834		121.781		N/A^e^		–0.196		.85		N/A	
	Purchase channels mentioned	–160.161		54.865		–0.124		–2.919		.004		1.183	
	Nonmonetary incentives mentioned	145.616		110.899		0.139		1.313		.19		7.343	
	No monetary incentives mentioned	104.164		97.565		0.106		1.068		.47		1.202	
	Age restrictions mentioned	26.925		37.420		0.031		0.720		.47		1.202	
	Flavors mentioned	–66.201		45.943		–0.060		–1.441		.15		1.128	
	Function of helping reduce stress mentioned	12.966		119.122		0.005		0.109		.91		1.300	
	Function of improving social skills mentioned	–38.685		126.013		–0.019		–0.307		.76		2.440	
	Function of entertainment	–21.014		136.283		–0.009		–0.154		.88		2.199	
	Engagement via posts encouraging reposts, comments, and likes	426.267		73.226		0.314		5.821		<.001		1.897	
	Engagement topics related to products	49.217		38.181		0.062		1.289		.20		1.518	
	Engagement topics related to brands	120.660		46.003		0.115		2.623		.009		1.249	
	Engagement topics related to current affairs	62.660		36.701		0.083		1.707		.09		1.527	
	Engagement topics related to interactive sweepstakes	35.513		84.653		0.034		0.420		.68		4.327	
**Model 2**													
	Constant	–31.261		17.061		N/A		–1.832		.07		N/A	
	Purchase channels mentioned	–11.961		7.593		–0.068		–1.575		.12		1.248	
	Nonmonetary incentives mentioned	43.244		14.885		0.301		2.905		.004		7.283	
	No monetary incentives mentioned	19.737		13.098		0.147		1.507		.13		6.449	
	Age restrictions mentioned	18.580		5.056		0.155		3.675		<.001		1.208	
	Flavors mentioned	–9.652		6.260		–0.064		–1.542		.12		1.153	
	Engagement via stories and ideas collecting	37.903		8.805		0.173		4.305		<.001		1.094	
	Engagement topics related to products	18.462		5.183		0.170		3.562		<.001		1.540	
	Engagement topics related to brands	30.974		6.098		0.214		5.079		<.001		1.209	
	Engagement topics related to soul soother or emotional topics	1.487		12.691		0.005		0.117		.91		1.207	
	Engagement topics related to current affairs	15.813		4.965		0.152		3.185		.002		1.539	
	Engagement topics related to interactive sweepstakes	17.967		11.083		0.126		1.621		.11		4.084	
	Salesmen images appeared in the posts	0.002		0.005		0.019		0.461		.65		1.182	
**Model 3**													
	Constant	579.228		130.244		N/A		4.447		<.001		N/A	
	Purchase channels mentioned	–17.749		44.922		–0.017		–0.395		.69		1.184	
	Nonmonetary incentives mentioned	–763.118		90.786		–0.880		–8.406		<.001		7.343	
	No incentives mentioned	–489.187		79.866		–0.603		–6.125		<.001		6.498	
	Age restrictions mentioned	5.712		31.559		0.008		0.181		.86		1.276	
	No health warnings mentioned	–68.884		75.751		–0.037		–0.909		.36		1.090	
	No product information mentioned	–74.531		32.205		–0.126		–2.314		.02		1.975	
	Function of entertainment	4.908		76.594		0.003		0.064		.95		1.036	
	Flavors mentioned	–64.256		39.541		–0.070		–1.625		.11		1.247	
	Engagement via stories and ideas collecting	90.489		51.911		0.068		1.743		.08		1.03	
	Engagement topics related to products	6.503		34.271		0.010		0.190		.85		1.824	
	Engagement topics related to brands	149.064		37.730		0.171		3.951		<.001		1.254	
	Engagement topics related to interactive sweepstakes	451.256		67.904		0.523		6.645		<.001		4.155	
	Engagement topics related to current affairs	22.219		30.368		0.035		0.732		.47		1.560	

^a^T: T-value.

^b^VIF: variance inflation factor.

^c^B: coefficient B.

^d^β: standardized coefficient beta.

^e^N/A: not applicable.

Contents including engagement via posts encouraging reposts, comments, and likes (*P<.*001), and engagement topics related to e-cigarette brands were positively correlated with the number of reposts (*P=.*009). Posts highlighting nonmonetary incentives (*P=*.004), posts with age-restriction statements (*P<.*001), engaging via stories and idea collection (*P<.*001), and engagement topics related to products *(P<.*001) and current affairs (*P=*.002) had a positive effect on the number of comments. Engagement topics related to brands (*P<.*001) or interactive sweepstakes (*P<.*001) had a more significant positive effect on the number of likes than those without. Posts that mentioned purchase channels significantly negatively affected the number of reposts (*P*=.004) of e-cigarette posts. Posts that did not mention any incentives had a negative impact on receiving likes of posts (*P*<.001). Posts that had no product information had a negative correlation with the number of likes (*P=*.02).

## Discussion

### Principal Findings

We analyzed Weibo posts by e-cigarette brands and gained valuable insight into how e-cigarette promotion information spreads and proliferates via social media. Weibo posts by 32 official e-cigarette accounts collectively received many likes, comments, and reposts, gaining widespread attention. Our results show that e-cigarette content exposure on social media may attract Weibo users’ attention and can successfully shape more positive attitudes toward e-cigarettes. Existing research has also shown that social media marketing of e-cigarettes has a particularly positive impact on teenagers’ attitudes and risk behavior [49,50], which may pose a threat and challenge to youth e-cigarette use and global public health.

### Marketing Tactics on Chinese Social Media

We observed several marketing tactics employed by e-cigarette companies on Chinese social media, documented as emphasizing attractive product features, using trendy characters, implicit promotions, downplaying health concerns, and engaging with Weibo users in various ways. These posts were visualized through pictures or videos, catering to teenagers’ reading habits and the demand for more impactful content [51,52]. The persuasive power of images was crucial in making e-cigarette-related information more attractive [42]. Few posts mentioned health warnings and age restrictions.

Highlighting attractive product characteristics was one of the most significant e-cigarette marketing tactics on Weibo. Approximately half of the Weibo posts emphasized product characteristics, particularly showcasing unique shapes and attractive packaging, which may appeal to teenagers and lower their perceptions of e-cigarette risks [38,39]. Even though only a small number of posts mentioned flavors, the promotion of flavors through beautifully designed posters and appealing flavor descriptions should be further monitored. This finding revealed that flavor features attract teenagers [22,40-42], as it is the most common reason for youth e-cigarette use [43]. In particular, some e-cigarette brands marketed flavors that represent Chinese culture, such as green tea. To the best of our knowledge, this is the first study to identify a marketing strategy that builds a responsible corporate image by promoting efforts to protect traditional Chinese culture. Among all the posts with images of people, fashion icons and ordinary people accounted for the largest proportion. The latest research suggests that attention should be paid to graphic characters in e-cigarette advertisements, as fashion models or ordinary people may glorify or normalize e-cigarette use among teenagers [53].

Even after online sales supervision was tightened in 2020 [19], a small number of e-cigarette companies continue to provide relevant promotional information on Weibo and continue to look for implicit promotion methods. The impact of nonmonetary incentives was uncertain in our findings. However, the existing published scoping reviews reveal that e-cigarette companies worldwide use monetary and nonmonetary incentives, such as promotional codes, discounts for new customers, coupons, and giveaways, in addition to e-cigarette advertising [44,54]. Double incentive strategies likely create excitement and reciprocity, making teenagers feel grateful toward the brand, thus promoting a positive perception of that e-cigarette brand [55].

The scarcity of age restrictions and health warnings in e-cigarette posts was common, potentially reducing Weibo users’ sense of caution. Previous studies have also highlighted the lack of emphasis on health issues and warnings by e-cigarette companies on social media [1,5,21,56,57]. Previous studies have found nicotine warnings of different designs could influence young people’s perception of harm and use intention to varying degrees [58]. In our study, we found relevant statements were in small print and vague, which might not draw enough attention or mislead teens. Therefore, warnings and statements should be carefully designed to alert or minimize appeal to teenagers. Additionally, such restrictions should be strictly regulated and monitored on social media.

One remarkable new finding regarding online engagement is that interactive sweepstakes on Weibo significantly expanded the influence of e-cigarette brands. In our study, the 3 posts with the highest reposts, comments, and likes all had interactive sweepstakes. Web-based contests and sweepstakes are also frequently utilized on social media platforms favored by young people in other countries [44,46]. A previous study emphasized the success of sweepstake-based marketing in enhancing user attention and engagement [59]. Reposts, comments, and likes are measures of user engagement [45,60] and indicators to increase the exposure of posts [61,62]. Web-based interactivity is a unique feature of social media that can create greater brand exposure and attention. Digital e-cigarette marketing involves various interactive methods to foster greater engagement. Our results show that various types of interaction positively impact the media effects of e-cigarette Weibo posts to varying degrees.

### The Establishment of Community Culture on Weibo

Based on the content analysis, our study found that e-cigarette companies constructed a unique culture to appeal to both e-cigarette users and potential users on Weibo. Interactive strategies on social media help e-cigarette brands establish an online presence that posits e-cigarettes as fashionable trend and lifestyle choice, potentially creating a community and identity for vapers and fostering a sense of belonging and loyalty. This conclusion is consistent with the existing research [63].

Our results uncovered many posts with hashtags, similar to the results of other studies [4,21]. Many e-cigarette Weibo accounts post hashtags with their brand name to build a vaping community associated with a specific e-cigarette brand. Using brand-specific hashtags can further construct social bonds and common beliefs around e-cigarettes [1,64]. Posts including hashtags unrelated to tobacco directly are common [4], and existing studies revealed vaping posts contained health lifestyle hashtags [65]; thus, teenagers may view specific hashtags posted by e-cigarette companies without searching for e-cigarette-related content, which can greatly expand a post’s reach. For example, one of the posts included hashtags such as #daretoimagine (“Gan Yu Xiang Xiang” in Chinese). Moreover, Chinese e-cigarette marketing on Weibo combines mainstream culture and subculture through hashtags related to current events. For example, some hashtags linked the new technology of e-cigarette products to China’s overall technological power. Through this marketing strategy, e-cigarette Weibo posts achieve broader coverage.

### Implications for Policy Making

China gradually strengthened regulations regarding e-cigarette marketing after this study was completed. The latest policy tightens restrictions on the manufacture and sale of e-cigarettes, specifically prohibiting the sale of flavored e-cigarettes, tobacco flavors, and private company atomized e-cigarettes [20]. After the ban, there has been a massive decline in the e-cigarette marketing strategies we observed in this study. However, more implicit posting, instead of direct promotion content on social media, may be a way to circumvent these restrictions. Until May 10, 2024, among all the 32 Weibo accounts of e-cigarette companies we investigated, 6 (18.9%) Weibo accounts continued updating posts on Weibo after the published ban in 2022 [20], and 1 (3.1%) e-cigarette company is still updating posts on Weibo as of April 2024. The content posted by these e-cigarette companies on Weibo did not directly promote e-cigarettes. Rather, most posts described e-cigarette flavors covertly via references to coffee, desserts, and other food-related items.

Our findings suggest that hidden online promotional content poses a significant threat and challenge to youth e-cigarette use and global public health. Thus, regulatory policies should be implemented to protect teenagers from marketing information regarding e-cigarettes and other tobacco products. Constant surveillance and nationwide legislation of internet marketing activities are essential to protect teenagers from ambiguous, inaccurate, or even deceptive information about e-cigarettes, as teenagers and other vulnerable populations are more susceptible to marketing claims [31,32,66]. There should be clear legal provisions that e-cigarette–related information must include explicit health claims and age restriction statements in large print [67,68]. Furthermore, more relevant health education activities and campaigns for teenagers should be conducted, and health communication media should give more prominence to issues related to e-cigarette prevention and control.

In response to the rapid growth of e-cigarette marketing, China has been intensifying its efforts to regulate e-cigarette internet marketing, which could serve as a reference for enhancing electronic cigarette oversight globally.

### Limitations

This study had several limitations that need to be addressed in future research. First, our study focused on the official Weibo accounts of e-cigarette brands, neglecting other stakeholders and social media platforms involved in e-cigarette social media marketing. Second, our data collection did not consider the possibility of data manipulation by e-cigarette companies. Third, although the existing research shows that e-cigarette marketing is mainly targeted toward teenagers, we could not confirm whether the e-cigarette Weibo marketing strategies are directly reflected in this age group due to the anonymity on social media. Direct evidence of the relationship between Weibo e-cigarette marketing and its impact on teenagers still needs to be teased out. Future research could gather data from more countries and platforms to conduct a more internationally comparative study.

### Conclusion

The findings reveal marketing tactics used by e-cigarette companies on Chinese social media, and their influence on the numbers of reposts, comments, and likes on posts. Despite nationwide regulations, e-cigarette companies are finding ways to strengthen their marketing presence on Weibo and indirectly target adolescents, often without health warnings or age restriction disclaimers. E-cigarette marketing on social media requires global attention and coordinated efforts. Our study provides insights into how to strengthen the supervision of invisible marketing on social media of e-cigarette companies.

## Appendix

Multimedia Appendix 1 Codebook and descriptive statistics of marketing characteristics of 863 posts from 32 selected Chinese e-cigarette Weibo accounts (October 1 to December 31, 2020).

## Abbreviations

ENDS: electronic nicotine delivery systems
PRISMA: Preferred Reporting Items for Systematic Reviews and Meta-Analyses
T: T-value
VIF: variance inflation factor
WHO: World Health Organization

## Data Availability

Data are available on reasonable request.
