# Supplementary material for: Social Media Marketing Strategies for Electronic Cigarettes: Content Analysis of Chinese Weibo Accounts
Source: J Med Internet Res. 2024 Nov 7;26:e51594. doi: 10.2196/51594 (PMC11582489; doi:10.2196/51594)
Supplement: Multimedia Appendix 1 [file jmir_v26i1e51594_app1.docx]

| Section | Theme | Items | Definition | Total sample (N=863) |
| --- | --- | --- | --- | --- |
| **Product and features** |  |  |  |  |
|  | Product characteristics |  |  |  |
|  |  | None |  | 414(48.0%) |
|  |  | Shape and packaging attractiveness | Highlight the shape of the product and/or attractive package. | 351(40.7%) |
|  |  | Flavors | Illustrate various attractive e-flavors. | 111(12.9%) |
|  |  | High quality and technique | Highlight that the e-cigarette product is of good quality and is made via high-technology | 48(5.6%) |
|  | Product function |  |  |  |
|  |  | Not mentioned |  | 775(89.8%) |
|  |  | Help quit smoking | Emphasize e-cigarettes can help cigarette users quit smoking | 2(0.2%) |
|  |  | Substitute for cigarettes | Highlight e-cigarettes can be a substitute for cigarettes | 8(0.9%) |
|  |  | Help reduce health risks | Propagate that e-cigarettes have fewer health risks compared to cigarettes | 3(0.3%) |
|  |  | Help reduce stress | Highlight e-cigarettes have the function of reducing stress | 10(1.8%) |
|  |  | Help improve social skills | Highlight one can show more social skills by using e-cigarettes | 27(3.1%) |
|  |  | Can make one appear trendy | Highlight one can be more fashionable using e-cigarettes | 74(8.6%) |
|  |  | Highlight entertainment features | Emphasize that e-cigarettes are playable and have entertainment functions | 23(2.7%) |
|  |  | Can improve social status | Explain that e-cigarette use has the function of improving social status | 2(0.2%) |
|  | Occupation of popular people in the image |  |  |  |
|  |  | Unable to judge |  | 85(9.85%) |
|  |  | Fashion icon | Depict fashion icons using/wearing e-cigarettes. | 81(9.4%) |
|  |  | Ordinary person | Depict ordinary people using/wearing e-cigarettes. | 78(9.0%) |
|  |  | Businessperson | Depict businessmen wearing e-cigarettes and/or their stories related to e-cigarettes. | 26(3.0%) |
|  |  | Sportsperson | Combine e-cigarettes with sportsmen in some activities. | 16(1.9%) |
|  |  | Celebrity | Relate celebrities to e-cigarettes/e-cigarette brands. | 13(1.5%) |
|  |  | Sales agent | Show salesmen introducing and/or promoting e-cigarettes. | 12(1.4%) |
|  |  | Health professional | Health professionals mentioned via images or videos, such as Zhong Nanshan, a respiratory medicine specialist in China. | 2(0.2%) |
| **Sales and promotion** |  |  |  |  |
|  | Information on product promotion |  |  |  |
|  |  | Not mentioned |  | 762(88.3%) |
|  |  | Monetary incentives | Promotional monetary information is included, such as discount coupons, two-for-one offers, and discount sales. | 10(1.2%) |
|  |  | Non-monetary incentives | Promotional non-monetary information is included, such as giveaways and free samples. | 83(9.6%) |
|  |  | Double incentives | Provide both monetary and non-monetary incentives in one post. | 8(0.9%) |
|  |  |  |  |  |
|  | Information on purchase channels |  |  |  |
|  |  | Not mentioned |  | 790(91.5%) |
|  |  | Mentioned | Information about offline stores, purchase links, and phone numbers included. | 73(8.5%) |
| **Social engagement** |  |  |  |  |
|  | Ways to interact |  |  |  |
|  |  | None |  | 315(36.5%) |
|  |  | Hashtags | Posts include hashtags to increase exposure and/or build an e-cigarette identity or community. | 538(62.3%) |
|  |  | Posts encouraging fans to repost, comment, and click likes | Encourage fans to repost, give comments, and click likes. | 41(4.8%) |
|  |  | Collect stories and ideas | Encourage fans to share their stories and/or ideas related to e-cigarettes. | 29(3.4%) |
|  | Interaction topics |  |  |  |
|  |  | Products | Encourage fans to share their opinions on e-cigarette product. | 392(45.4%) |
|  |  | Current events | Link products to current events. | 182(21.1%) |
|  |  | Interactive sweepstakes | Encourage fans to participate in sweepstakes by following a Weibo account and/or reposting and/or giving comments and/or clicking likes. | 75(8.7%) |
|  |  | Brands | Encourage fans to share their opinions on e-cigarette brands. | 73(8.5%) |
|  |  | ‘Soul-soother’ or emotional topics | Give life insights and/or life philosophy about emotional topics. | 15(1.7%) |
|  |  | Weibo accounts promotion | Use words and/or pictures to promote a Weibo account. | 1(0.1%) |
|  |  |  |  |  |
| **Restrictions and warnings** |  |  |  |  |
|  | Claims for restricting minors’ purchase or use |  |  |  |
|  |  | Not mentioned |  | 687(79.6%) |
|  |  | Use restricted but purchase unlimited | State minors are prohibited from using e-cigarettes. | 130(15.1%) |
|  |  | Purchase restricted but use unlimited | State minors are prohibited from purchasing e-cigarettes. | 6(0.7%) |
|  |  | Both restricted | State minors are prohibited from using or purchasing any e-cigarette products. | 40(4.6%) |
|  | Information on health warnings |  |  |  |
|  |  | Not mentioned |  | 839(97.2%) |
|  |  | Mentioned | Health warnings related to e-cigarette use. | 24(2.8%) |
